# CTGF facilitates cell‐cell communication in chondrocytes via PI3K/Akt signalling pathway

**DOI:** 10.1111/cpr.13001

**Published:** 2021-02-01

**Authors:** Zuping Wu, Chenchen Zhou, Quan Yuan, Demao Zhang, Jing Xie, Shujuan Zou

**Affiliations:** ^1^ State Key Laboratory of Oral Diseases West China Hospital of Stomatology Sichuan University Chengdu China; ^2^ National Clinical Research Center for Oral Diseases West China Hospital of Stomatology Sichuan University Chengdu China

**Keywords:** chondrocytes, Connexin43, CTGF, gap junction intercellular communication

## Abstract

**Purposes:**

Gap junction intercellular communication (GJIC) is essential for articular cartilage to respond appropriately to physical or biological stimuli and maintain homeostasis. Connective tissue growth factor (CTGF), identified as an endochondral ossification genetic factor, plays a vital role in cell proliferation, migration and adhesion. However, how CTGF regulates GJIC in chondrocytes is still unknown. This study aims to explore the effects of CTGF on GJIC in chondrocytes and its potential biomechanism.

**Materials and methods:**

qPCR was performed to determine the expression of gene profile in the CCN family in chondrocytes. After CTGF treatment, CCK‐8 assay and scratch assay were performed to explore cell proliferation and migration. A scrape loading/dye transfer assay was adopted to visualize GJIC in living chondrocytes. Western blot analysis was done to detect the expression of Cx43 and PI3K/Akt signalling. Immunofluorescence staining was used to show protein distribution. siRNA targeting CTGF was used to detect the influence on cell‐cell communication.

**Results:**

The CTGF (CCN2) was shown to be the highest expressed member of the CCN family in chondrocytes. CTGF facilitated functional gap junction intercellular communication in chondrocytes through up‐regulation of Cx43 expressions. CTGF activated PI3K/Akt signalling to promote Akt phosphorylation and translocation. Suppressing CTGF also reduced the expression of Cx43. The inhibition of PI3K/Akt signalling decreased the expressions of Cx43 and thus impaired gap junction intercellular communication enhanced by CTGF.

**Conclusions:**

For the first time, we provide evidence to show CTGF facilitates cell communication in chondrocytes via PI3K/Akt signalling pathway.

## Graphical abstract

Exogenous CTGF increased the expression of gap junction forming protein, Cx43, and enhanced functional gap junction channels to promote the cell‐to‐cell communication in chondrocytes. CTGF activated the PI3K/Akt signalling pathway and induced the nuclear translocation of p‐Akt to regulate the Cx43 expression and the following gap junction intercellular communication.

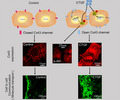

## 1 INTRODUCTION

The articular cartilage is located on the surface of the connected bones, which promotes the joint to achieve three‐dimensional movement with low‐friction. This tissue contains extracellular matrix (ECM), mainly composed of type II collagen and proteoglycans. Chondrocytes are highly specialized cells embedded in the ECM, which are essential for locomotion and postnatal growth. Chondrocytes account for less than 10% of the total volume of articular cartilage, but are responsible for cartilaginous matrix maintenance, formation and repair throughout adulthood.^1^ Proper cell‐to‐cell communication is essential for tissues to respond appropriately to physical or biological stimuli and maintain tissue homeostasis with activation of signalling pathways such as proliferation, differentiation and death.^2^ Gap junction proteins have significant roles in such cellular communication, including connexins (Cxs) and pannexins (pxs).^2^, ^3^, ^4^

The connexons are formed by the oligomerization of six connexins, function as hemichannels, regulating the release of small signalling molecules such as calcium, other ions, ATP and prostaglandins between cells and extracellular environment.^5^, ^6^ In addition, two connexons from adjacent cells dock with each other to form gap junctions, thereby providing a channel for direct exchange of small signalling molecules (<1 kDa) between communicating cells. Cx43 is the most widely expressed connexins in these tissues, which are thought to play an important role in organogenesis and homeostasis. Cx43 was located at the edge of the cell and expressed in each layer of articular cartilage, while mainly expressed in the superficial zone of the growth plate.^7^, ^8^, ^9^ Connexins dysfunction may cause joint diseases such as rheumatoid arthritis (RA), osteoarthritis (OA).^7^, ^10^

Connective tissue growth factor (CTGF), known as CCN2, is a 38kDa extracellular matrix protein, a member of CCN (cellular communication network factor) protein family. These proteins are made up of five domains, including secretory signal peptide (SP), insulin‐like growth factor‐binding protein (IGFBP) module, von Willebrand factor type C repeat (VWC), thrombospondin type 1 repeat (TSP1), and C‐terminal (CT) module.^11^ CTGF, identified as an endochondral ossification genetic factor, plays vital roles in regulating various cellular functions, including proliferation, migration, adhesion, survival, differentiation and synthesis of ECM proteins in skeletal development.^12^, ^13^, ^14^ CTGF participates in a variety of biological processes and plays multiple roles, such as angiogenesis, chondrogenesis, osteogenesis, wound healing, fibrosis and tumour formation.^13^, ^14^, ^15^ In addition, applying CCN2 together with gelatin hydrogel to cartilage defects can repair articular cartilage.^16^ Transfection with siRNA targeting CTGF ribonucleic acid in cardiomyocytes reduced Cx43 expression.^17^ However, the relationship between CTGF and cell‐cell communication is not yet known and needs be further investigated.

In our study, we used recombinant CTGF to explore the relationship between CTGF and the gap junction in chondrocytes. We examined the effects of CTGF on intercellular communication, Cx43 expression, cell proliferation and migration. Furthermore, we demonstrated that CTGF induced Cx43 formation through the phosphatidylinositol 3‐kinase/Akt (PI3K/Akt) signalling pathway.

## 2 METHOD AND MATERIALS

### 2.1 Preparation of tissues and cells

The animal materials used in this study meet the requirements of ethical principles, and all protocols have been reviewed and approved by the institutional review board of our hospital (IRB, institutional review board of West China Stomatological Hospital, number: wchsirb‐D‐2017‐029). Articular cartilage was isolated from newborn C57BL mice by using the enzymatic digestion method. The articular cartilages were cut into pieces and trypsinized with 0.25% protease solution dissolved in Dulbecco's modified Eagle's medium (high‐glucose DMEM, 0.1 mmol/l non‐essential amino acids, 4 mmol/l L‐glutamine, 1% penicillin‐streptomycin solution, HyClone, Logan, UT, USA) for 30 minutes at 37°C, and then was digested with 0.2% type II collagenase (sigma) at 37°C for 12 hours. After centrifugation with 8 minutes at 1000 rpm, the chondrocytes were seeded onto 25‐cm^2^ cell culture flasks in DMEM supplemented with 10% foetal bovine serum (FBS) and 1% penicillin/streptomycin and incubated with 5% CO_2_ at 37°C. The culture media were changed every 2 days.

### 2.2 Quantitative real‐time PCR (qPCR)

Total RNA was extracted from primary articular chondrocytes with the RNeasy Plus Mini Kit (Qiagen, CA) according to the manufacturer's protocol. The extracted RNA samples were dissolved in RNase‐free water and concentration of RNA samples was quantified by the NanoDropR spectrophotometer (Nano Spectrophotometer 2000c, Thermo Fisher Scientific). Reverse transcription was performed with the cDNA synthesis kit (K1621‐RevertAid, Mbi, MD) and Quantitative real‐time PCR was conducted with the SYBR Premix ExTaq II PCR Kit (TAKARA, Shiga, Japan) on a Bio‐rad CFX manager instrument according to the manufacturer's protocol. The quantified relative expression of the gene of interest was normalized to GAPDH housekeeping gene by using the 2^−ΔΔCt^ method. The primer sequences were shown in Table 1.

**TABLE 1:** Sequences of primer pairs of housekeeping and CCN family genes in chondrocytes for qPCR

Protein name	Gene name/gene ID	Primer pairs
Glyceraldehyde‐3‐phosphate dehydrogenase (GAPDH)	GAPDH (101bp)	Forward: AGGTTGTCTCCTGCGACTTCA
	(NM_001289726.1)	Reverse: CCAGGAAATGAGCTTGACAAA
Cellular communication network factor family member 1 (CCN1)	CCN1 (121bp)	Forward: TTGACCAGACTGGCGCTCTC
	(NM_010516.2)	Reverse: TAGCGCAGACCTTACAGCAG
Cellular communication network factor family member 2 (CCN2)	CCN2 (110bp)	Forward: AGAACTGTGTACGGAGCGTG
	(NM_010217.2)	Reverse: GTGCACCATCTTTGGCAGTG
Cellular communication network factor family member 3 (CCN3)	CCN3 (100bp)	Forward: CTGAGATGAGACCCTGTGACC
	(NM_010930.5)	Reverse: TTGTCTCCCTCTGGAACCAT
Cellular communication network factor family member 4 (CCN4)	CCN4 (117bp)	Forward: AATAGGAGTGTGTGCACAGGT
	(NM_018865.3)	Reverse: GTGCCATCAATGCAGGTACA
Cellular communication network factor family member 5 (CCN5)	CCN5 (143bp)	Forward: CCTCAGCCCAAGGACACCAA
	(NM_016873.2)	Reverse: TCGGTTCTGGTTGGATACTCG
Cellular communication network factor family member 6 (CCN6)	CCN6 (139bp)	Forward: GCAAAGTCTGTGCCAAGCAA
	(NM_001127376.1)	Reverse: CCAACTGCCACAAGATATGCG
Gap junction protein alpha 1 (GJA1)	Cx43 (126bp)	Forward: TGCACCTGGGGTGTTCATTT
	(NM_010288.3)	Reverse: GCCGCCTAGCTATCCCAAAA

### 2.3 Cell counting kit‐8 assay

To examine cell proliferation induced by CTGF (0 206 317, PeproTech), the chondrocytes were seeded onto a 96‐well plate at a density of 2000 cells per well. After being reached to adherence in 12 hours, chondrocytes were treated with different concentrations of CTGF and incubated for 24 hours. Each well was then supplemented with 20 μL of cell counting kit‐8 (CCK‐8) reaction solution for 2 hours incubation. The absorbance was measured via the CCK‐8 assay in accordance to the manufacturer's manual.

### 2.4 CTGF Small interfering RNA (siRNA) transfection

Cells were seeded onto 35 mm cell culture dishes for 12 hours and transiently transfected with specific siRNA oligonucleotides (hanbio, Shanghai, China) using Lipofectamine RNAiMAX (Invitrogen, Burlington, ON, Canada). These sequences were listed as follows: sense, 5′‐GGAAGAUGUACGGAGACAUTT‐3′; antisense, 5′‐AUGUCUCCGUACAUCUUCCTT‐3′. A vacant RNA carrier was applied as the scrambled group. All experiments were repeated thrice. After transfection for 48 hours, scrape loading and dye transfer (described below) were taken to visualize cell‐cell communication, and RNA samples and protein samples were extracted to analyse the CTGF and Cx43 expression by qPCR and western blot.

### 2.5 Western blot analysis

The expression levels of Cx43, CTGF, essential proteins (AKT, p‐AKT) involved in the PI3k/AKT signalling were analysed by Western blot. Briefly, chondrocytes were seeded at a density of 2 × 10^5^ cells/well and cultured with DMEM containing 10% FBS. After seeding, cells were starved using 2% FBS DMEM for 12 hours. After starvation, the culture medium was changed to 1% fresh FBS DMEM containing various concentrations of CTGF (10, 20, 50 and 100 ng/mL) and PI3K inhibitor LY294002 (10, 15 μmol/L) with 1% penicillin‐streptomycin incubated for different times to investigate the effect of CTGF on protein expressions in chondrocytes. Subsequently, total cellular protein was prepared by lysing the cells with RIPA lysis buffer (P0013B, Beyotime, Shanghai, China) containing 2% PMSF (P7626, Sigma) protease inhibitor in an ice bath. The protein concentrations of samples were evaluated by BCA assay (P0010, Beyotime, Shanghai, China). Protein samples were separated through 10% SDS‐PAGE and transferred to PVDF membranes. The membranes were blocked with 5% skim milk for 1 hour, then incubated with primary antibodies against β‐actin (anti‐mouse, sc‐47778, Santa Cruz Biotechnology, Cambridge, UK), Cx43 (anti‐rabbit, ab11370, Abcam, Cambridge, UK), Akt (anti‐rabbit, 342529, zen‐bio, China), phosphorylated Akt (anti‐rabbit, 340705, zen‐bio, China) and CTGF (anti‐rabbit, 342529, zen‐bio, China) at 4°C overnight, washed with TBST and incubated with corresponding anti‐mouse or anti‐rabbit secondary antibodies for 2 hours at room temperature (1:5000 dilution). The immunocomplexes were visualized with using a Immobilon^®^ Western (P90719, Millipore). The protein expression levels were analysed with Image J software (NIH, Bethesda, MD) and normalized against β‐actin.

### 2.6 Scanning electron microscopy (SEM)

Chondrocytes were seeded onto 35 mm cell culture dishes with 70% confluence. After incubating with CTGF or LY294002 for 12 hours, the culture medium was discarded and replaced by 2.5% glutaraldehyde for fixing for 2 hours. Besides, chondrocytes were seeded onto 35 mm cell culture dishes for transfection with 50 nmol/L siRNA that targeting CTGF gene. After 48 hours, the culture medium was also discarded and replaced by 2.5% glutaraldehyde for fixing for 2 hours. Then these cells were subsequently dehydrated with graded ethanol at concentration from 30%, 50%, 70%, 80%, 90%, to 100% for 15 minutes at each level. Subsequently, the samples were coated with a gold layer and then ready to be visualized for cell junctions by SEM.

### 2.7 Scratch assay

After articular chondrocytes were grown to a full confluent monolayer in a 6‐well plate, we applied a thin scratch in the middle of the cell layer by using a 200 μL sterile tip. After washing with PBS to discard cell debris, the attached cells were cultured in a basal medium with CTGF (10, 20, 50 and 100 ng/mL) for 12 and 24 hours. The cell migration and proliferation were captured at 0, 12 and 24 hours after scraping through phase‐contrast microscopy. The mobility ratio was evaluated by migrated cell area/scraped area by Image J.

### 2.8 Scrape loading and dye transfer

The functional gap junctions between chondrocytes were evaluated with the scrape loading/dye transfer (SL/DT) technique. Lucifer yellow (LY) dye was introduced by scraping a monolayer of cells and transferred from the dye‐loaded cells to adjacent cells through functional gap junctions. Until the chondrocytes grew to a full confluence in cell culture dishes, the medium was discarded and then cells were rinsed with Ca^2+^‐Mg^2+^‐PBS three times and scraped using a surgical blade, followed by the addition of 1 mg/mL Lucifer Yellow solution. After a 7‐10 minutes incubation at room temperature, the chondrocytes were rinsed three times with PBS to remove all extracellular dye and fixed by adding 4% paraformaldehyde (The fixation step can be removed when aiming to see the LY dye transmission in living cells). We then monitored LY dye transfer by confocal laser scanning microscopy (CLSM, Olympus, FV3000, Japan) to evaluate intercellular communication.

### 2.9 Immunofluorescence and confocal laser scanning microscopy (CLSM)

Chondrocytes were seeded and cultured in observation dishes specified for confocal laser microscopy (CLSM) for 12 hours. The effect of CTGF and LY294002 on the expression of Cx43 and p‐AKT was detected in chondrocytes by CLSM. Chondrocytes were firstly treated with 50 ng/mL CTGF for 2 and 12 hours with presence and absence of 10 μmol/L LY294002 to detect the expression of p‐AKT and Cx43, respectively. The culture medium was discarded and cells were rinsed with PBS three times and then fixed with 4% cold paraformaldehyde solution, permeabilized with 0.5% Triton X‐100 (Beyotime, China, Shanghai) for 10 minutes, and blocked with 5% BSA for 1 hour. After being washed with PBS three times, primary antibodies against Cx43 (1:300, Abcam, Cambridge, UK) and p‐AKT (1:200, 340705, zen‐bio, China) were added into the dishes and incubated at 4°C overnight, respectively. A secondary antibody conjugated to AlexaFluor647 (ab150075, Abcam) was used to incubate the samples for 2 hours. DAPI (D9542, Sigma, USA) and phalloidine (6 μmol/L, Invitrogen, CA) were applied to stain the nuclei and cytoskeleton. The cells then were observed with CLSM. All experiments were repeated at least three times.

### 2.10 Statistical analysis

Experiments were performed in triplicate. Quantitative results are presented as the mean ± standard deviation (SD) and plotted with (GraphPad Prism Inc, San Diego, CA). Data were assessed through one‐way ANOVA followed by Tukey's protected least‐significant difference post hoc test for multiple comparisons. *P* value < .05 was considered to be statistically significant.

## 3 RESULTS

### 3.1 CTGF promotes intercellular communication and gap junction formation in chondrocytes

**FIGURE 1:**
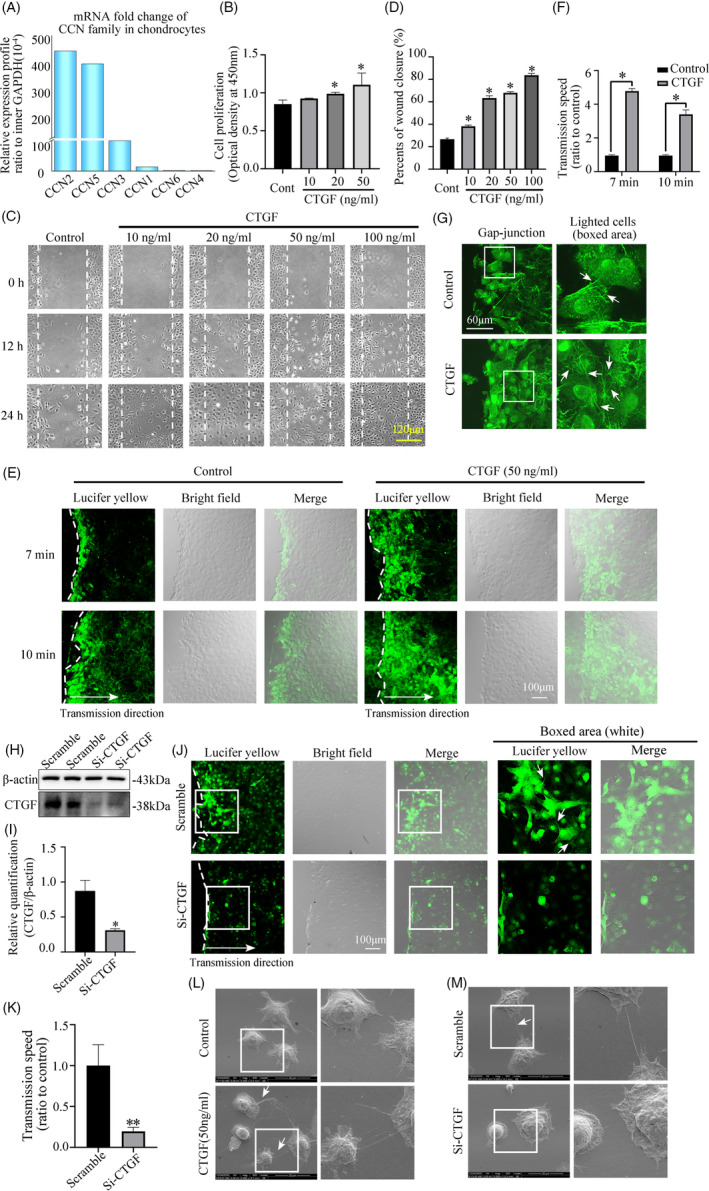
CTGF promotes cell‐to‐cell comunication in chondrocytes. A, Quantitative real‐time PCR showing the highest gene expression of CCN2 (CTGF) in CCN family. Relative gene profiles of CCN family were calculated by normalizing to internal GAPDH. Data were derived from the three independent experiments (n = 3). B, CCK‐8 assay showing increased cell proliferation of chondrocytes induced by CTGF. Data were derived from the three independent experiments (n = 3). *Significant difference was in comparison to the control group. Cont = control. C, Scratch wound closure assay was performed and captured by phase‐contrast microscopy. Data were based on three independent experiments (n = 3). D, Quantification was done to access the wound closure in Figure 1C. *Significant difference was in comparison to the control group. Cont = control. E, The scrape loading/dye transfer (SL/DT) assay showing the increased cell‐to‐cell transmission of Lucifer yellow through gap junction in chondrocytes induced by CTGF (50 ng/mL) within 7 and 10 min by CLSM. The results were derived from three independent experiments (n = 3). F, Quantification was done to access the gap junction formation in Figure 1E. *Significant difference was in comparison to the control group. G, Gap junction formations in living chondrocytes were shown to be increased in the CTGF group (50 ng/mL) by CLSM after Lucifer yellow loading at 1 mg/mL. The dye transfer images were collected at 10 min after Lucifer yellow loading. The results were based on three independent experiments (n = 3). The white arrows indicated the lightened gap junction channels. H, Western blot showing that small interfering RNA targetting CTGF at 50 nmol/L at 48 h inhibited the CTGF expression successfully at protein level. Scramble means scrambled control group. Si‐CTGF means the small interfering RNA targetting CTGF group. The data were based on three independent experiments (n = 3). I, Quantification was performed to show the protein changes of in Figure 1H. The statistical analyses were based on three independent experiments (n = 3). *P < .05. J, Gap junction formations in living chondrocytes were reduced in the siRNA group at 50 nmol/L siRNA at 48 h by CLSM after Lucifer yellow loading at 1 mg/mL. The dye transfer images were collected at 10 min after Lucifer yellow loading. The results were derived from three independent experiments (n = 3). The white arrows indicated the lightened gap junction channels. K, Quantification was done to access the gap junction formation in Figure 1J. **P < .01. L, Representative changes of intercellular connection in chondrocytes in response to CTGF (50 ng/mL) via SEM at 12 h treatment. The white arrows represent cell‐cell connecting junctions among chondrocytes. White boxed areas in the first column were zoomed in and displayed in the second one. The images were derived from three independent experiments (n = 3). M, Representative changes of intercellular connection in chondrocytes with Si‐CTGF (siRNA) via SEM at 48 h. White boxed areas in the first column were zoomed in and displayed in the second one. The white arrows represent cell‐cell connecting junctions among chondrocytes. The images were derived from three independent experiments (n = 3)

To begin with, we investigated the gene expressions of CCN family in primary chondrocytes. The CCN family comprises six members with a similar mosaic primary structure. They are Cyr61/CCN1, CTGF/CCN2, Nov/CCN3, WISP1/CCN4, WISP2/CCN5 and WISP3/CCN6.^13^ Our qPCR results indicated that mRNA of CTGF (CCN2) ranked No.1 in primary chondrocytes (Figure 1A).

Subsequently, CCK‐8 assay confirmed that the cell proliferation was also enhanced with the stimulation of CTGF in a dose‐dependent manner at 24 hours (Figure 1B). Then the scratch wound closure assay was applied to access the migration rates of chondrocytes at 12 and 24 hours after CTGF treatment with different concentrations. The analysis of the captured images indicated that cell migration was significantly accelerated in the CTGF group compared with the control group (Figure 1C&D). Intercellular communication was closely correlated with cell proliferation and migration due to the requirement of full cell confluence. Thus, we then explored the influence of CTGF on the gap junction formations in chondrocytes. With the scrape loading/dye transfer assay, the CTGF‐treated group showed higher ability to transfer Lucifer yellow (LY) dye between chondrocytes when compared with the control group (Figure 1E&F). Chondrocytes extended many cytoplasmic cilia to neighbouring cells to establish intercellular communication. The entire cell including nuclei was illuminated by Lucifer yellow, forming multiple transmission chains to transfer yellow fluorescent molecules to a farther distant cell.^18^, ^19^ Our results confirmed that CTGF promoted this gap junction formation in chondrocytes (Figure 1G). Besides, western blot showed siRNA transfection targeting CTGF successfully inhibited the expression of CTGF protein in chondrocytes (Figure 1H&I). Then siRNA in chondrocytes also reduced the ability of Lucifer yellow transmission when compared to the scrambled group (Figure 1J&K). To visualize the impact of CTGF on intercellular links among chondrocytes, the cells were then scanned by SEM. CTGF stimulation for 12 hours promoted the formation of the cell‐cell connections and induced network communication between chondrocytes (Figure 1L). Accordingly, siRNA of CTGF in chondrocytes for 48 hours showed the lower ability of connecting with other cells (Figure 1M).

### 3.2 CTGF promotes gap junction formation through the increase of Cx43 protein in chondrocytes

**FIGURE 2:**
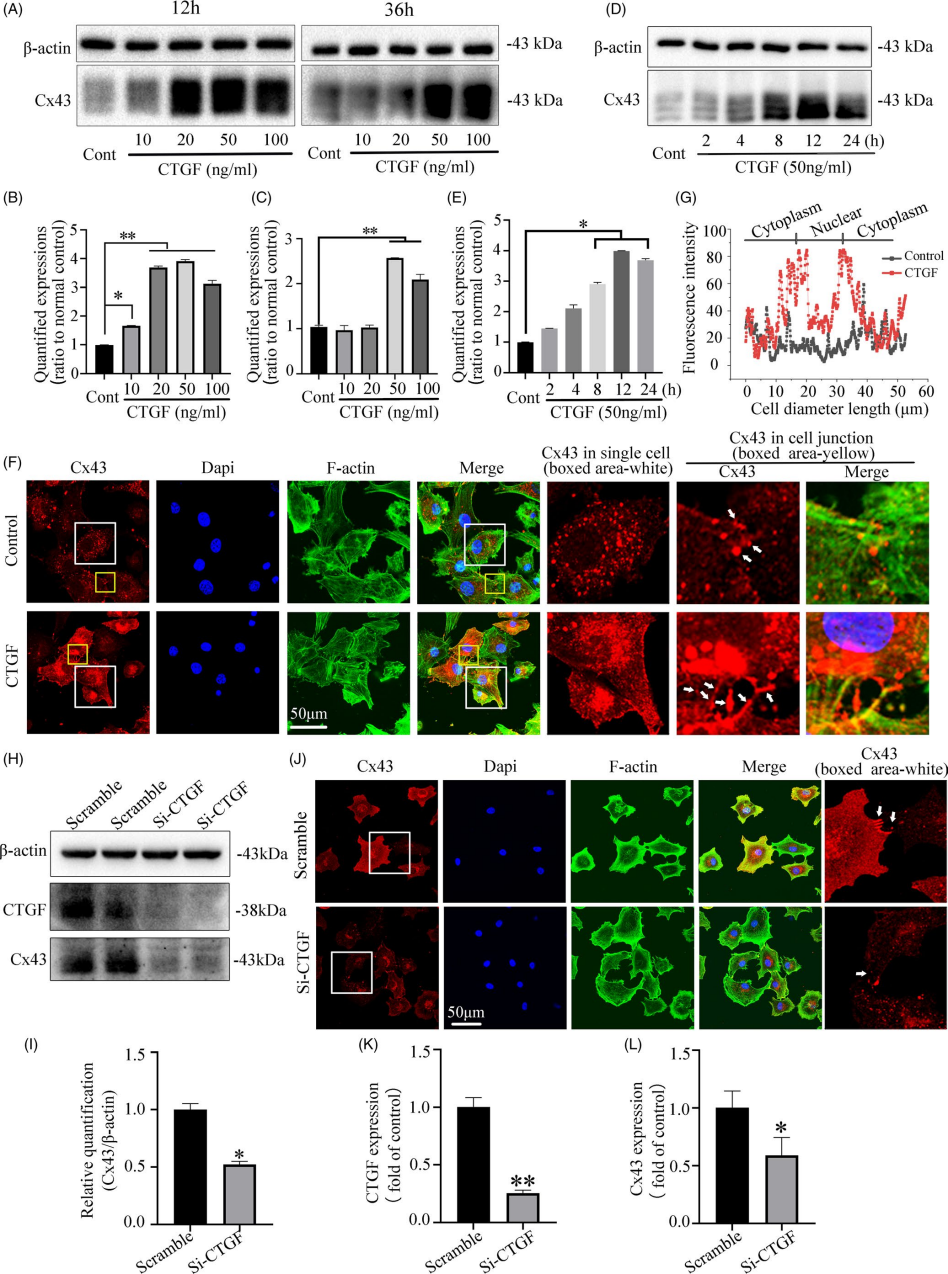
The CTGF‐enhanced gap junction formation is dependent on the increase of Cx43. A, Western blot showing that CTGF promoted Cx43 expression in a dose‐dependent manner. Results shown were collected at 12 h and 36 h after CTGF treatment. The data were based on three independent experiments (n = 3). B‐C, Quantification was performed to show the protein changes of Cx43 at 12 h (B) and 36 h (C) in Figure 2 A. The statistical analyses were based on three independent experiments (n = 3). *P < .05; **P < .01. D, Western blot showing that CTGF (50 ng/mL) promoted Cx43 expression in a time‐dependent manner. The data ware based on three independent experiments (n = 3). *P < .05. E, Quantification was performed to show the protein changes of Cx43 in Figure 2D after CTGF treatment (50 ng/mL). The statistical analyses were based on three independent experiments (n = 3). *P < .05. F, Representative IF staining by CLSM showing the enhanced expression and distribution of Cx43 in chondrocytes induced by CTGF (50 ng/mL). Cytoskeleton was stained with FITC‐phalloidin (green) and nuclei were stained with Dapi (blue). White arrows showed Cx43 accumulation was along gap junction. The results were derived from three independent experiments (n = 3). G, Fluorescence optical density (OD) assay was performed to show the distribution of Cx43 cross the cell body in Figure 2F. H, Western blot showing that siRNA of CTGF in chondrocytes reduced Cx43 expression within 48 h. The data were based on three independent experiments (n = 3). I, Quantification was performed to show the protein changes of Cx43 in Figure 2H. The statistical analyses were based on three independent experiments (n = 3). *P < .05. J, Representative IF staining by CLSM showing the decreased expression of Cx43 in siRNA of CTGF‐treated chondrocytes. Cytoskeleton was stained with FITC‐phalloidin (green) and nuclei were stained with dapi (blue). White arrows showed Cx43 accumulation was along gap junction. The results were derived from three independent experiments (n = 3). K, qPCR showed reduced CTGF gene expression after siRNA of CTGF transfection in chondrocytes. The results were derived from three independent experiments (n = 3). **P < .01. L, qPCR showed reduced Cx43 gene expression after siRNA of CTGF transfection in chondrocytes. The results were derived from three independent experiments (n = 3). *P < .05

Cx43, one of gap junction proteins, is widely expressed in 80%‐100% of chondrocytes in each zone.^20^ Our previous report had confirmed that the mRNA level of Cx43 was the highest among the connexin family in the primary chondrocytes.^19^ In order to explore the effect of CTGF on Cx43, we performed western blot and immunofluorescence. Western blot assay confirmed that the increase of Cx43 protein was in line with the increased concentrations of CTGF (Figure 2A). In addition, quantification confirmed that the expression of Cx43 after CTGF treatment (50 ng/mL) was much higher than that of the control group at both 12 hours (Figure 2B) and 36 hours (Figure 2C). We further examined the expressions of Cx43 at different time points induced by CTGF at 50 ng/mL. The results showed that Cx43 was increased in a time‐dependent manner (Figure 2D&E). To further explore the distribution of Cx43 in CTGF‐induced chondrocytes, immunohistochemical staining was performed and it was found that Cx43 is mainly located in the cytoplasm, cell membrane and intercellular connection (Figure 2F). After treatment with 50 ng/mL CTGF, we found that Cx43 was accumulated increasingly in the cytoplasm, cell membrane and gap junction plaques between cells which were characterized by continuous dotted lines (Figure 2F‐boxed area, yellow). Further fluorescent qualification also confirmed that the expression of Cx43 in the CTGF‐treated group was significantly enhanced (Figure 2G). Furthermore, suppressing CTGF expression by siRNA reduced the expression of Cx43 protein when compared to the scrambled group (Figure 2H&I). CLSM also showed siRNA in chondrocytes reduced the Cx43 expression in cell membranes and cell‐cell connection (Figure 2J). At mRNA levels, inhibiting the levels of CTGF also reduced the expression of Cx43 mRNA (Figure 2K&L).

### 3.3 CTGF triggers PI3K/AKT signalling to mediate the expression of connexin43

**FIGURE 3:**
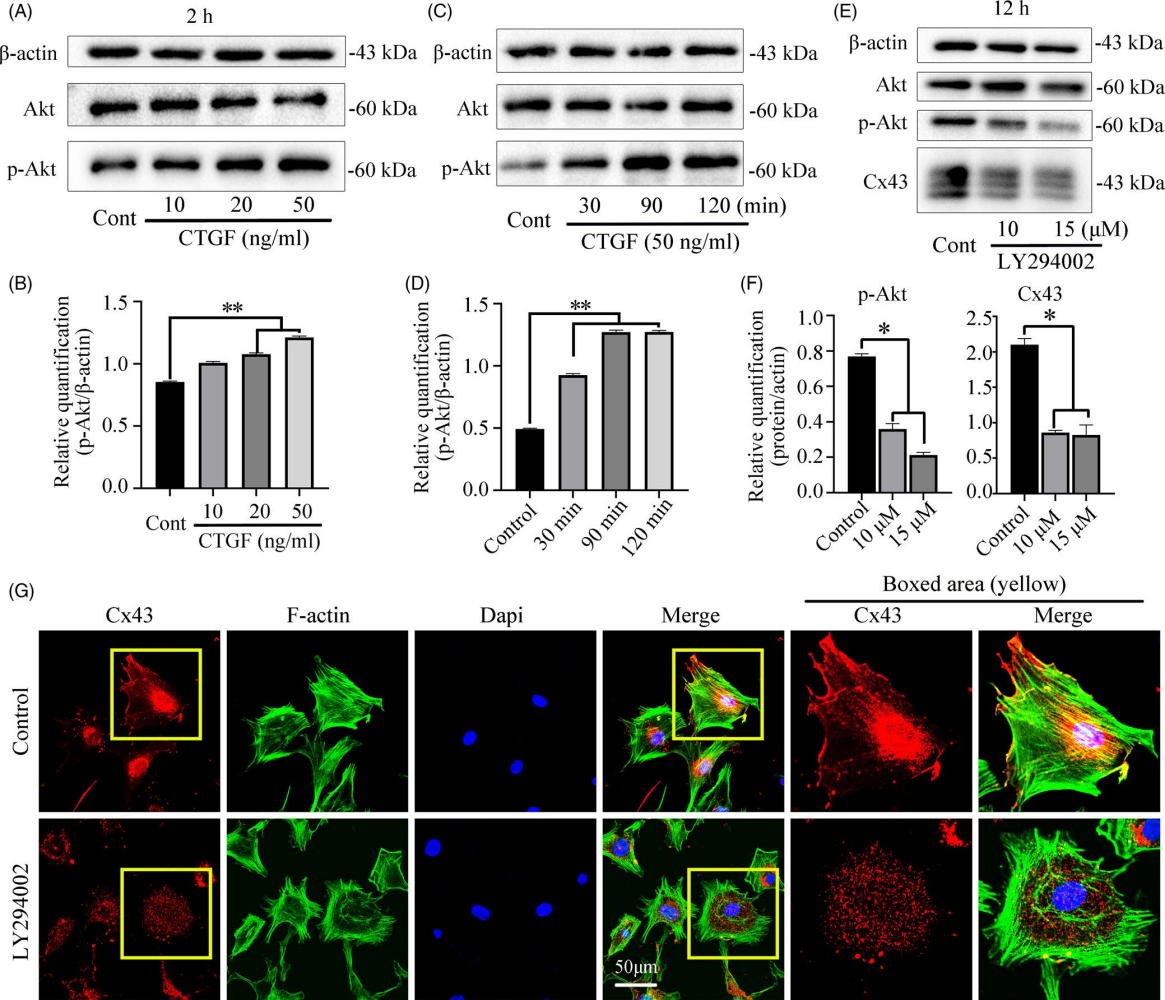
CTGF triggers PI3K/AKT signalling and inhibition of PI3K/AKT signalling reduced Cx43 expression. A, Western blot showing the expression of Akt, p‐Akt induced by CTGF for 2 h. The results were based on three independent experiments (n = 3). B, Quantification was performed to confirm the protein changes in Figure 3. A. The statistical analyses were based on three independent experiments (n = 3). **p < .01. C, Western blot showing the expression of Akt, p‐Akt induced by CTGF (50 ng/mL) for 30, 90 and 120 min. The results were based on three independent experiments (n = 3). D, Quantification was performed to confirm the protein changes in Figure 3. C. The statistical analyses were based on three independent experiments (n = 3). **P < .01. E, Western blot showing the expressions of Akt, p‐Akt and Cx43 after treatment with LY294002 (PI3k inhibitor, 10μM and 15μM) for 12 h. The results were based on three independent experiments (n = 3). F, Quantification was performed to confirm the protein changes in Figure 3. E. The statistical analyses were based on three independent experiments (n = 3). *P < .05. G, Representative IF staining by CLSM showing the inhibitory effect of LY294002 (10 μmol/L) on Cx43 in chondrocytes at 12h. Cytoskeleton, green; Cx43, red; nuclei, blue. The results were derived from three independent experiments (n = 3)

The PI3K/Akt signalling pathway was an important regulator of cellular proliferation and differentiation in chondrocytes.^21^, ^22^ We found that CTGF could activate the protein level of p‐Akt in a dose‐dependent manner (Figure 3A&B). At 50 ng/mL, CTGF increased the expressions of p‐Akt in a time‐dependent manner (Figure 3C&D). We then used LY294002, a specific PI3K inhibitor for PI3K/Akt signalling and found that LY294002 reduced the expression of p‐Akt at 10 and 15 μmol/L. Furthermore, we found that LY294002 could reduce the expression of Cx43 at 10 and 15 μmol/L (Figure 3E&F). The immunofluorescence assay further confirmed LY294002 could specifically reduce the expression of Cx43 compared with the control group (Figure 3G).

**FIGURE 4:**
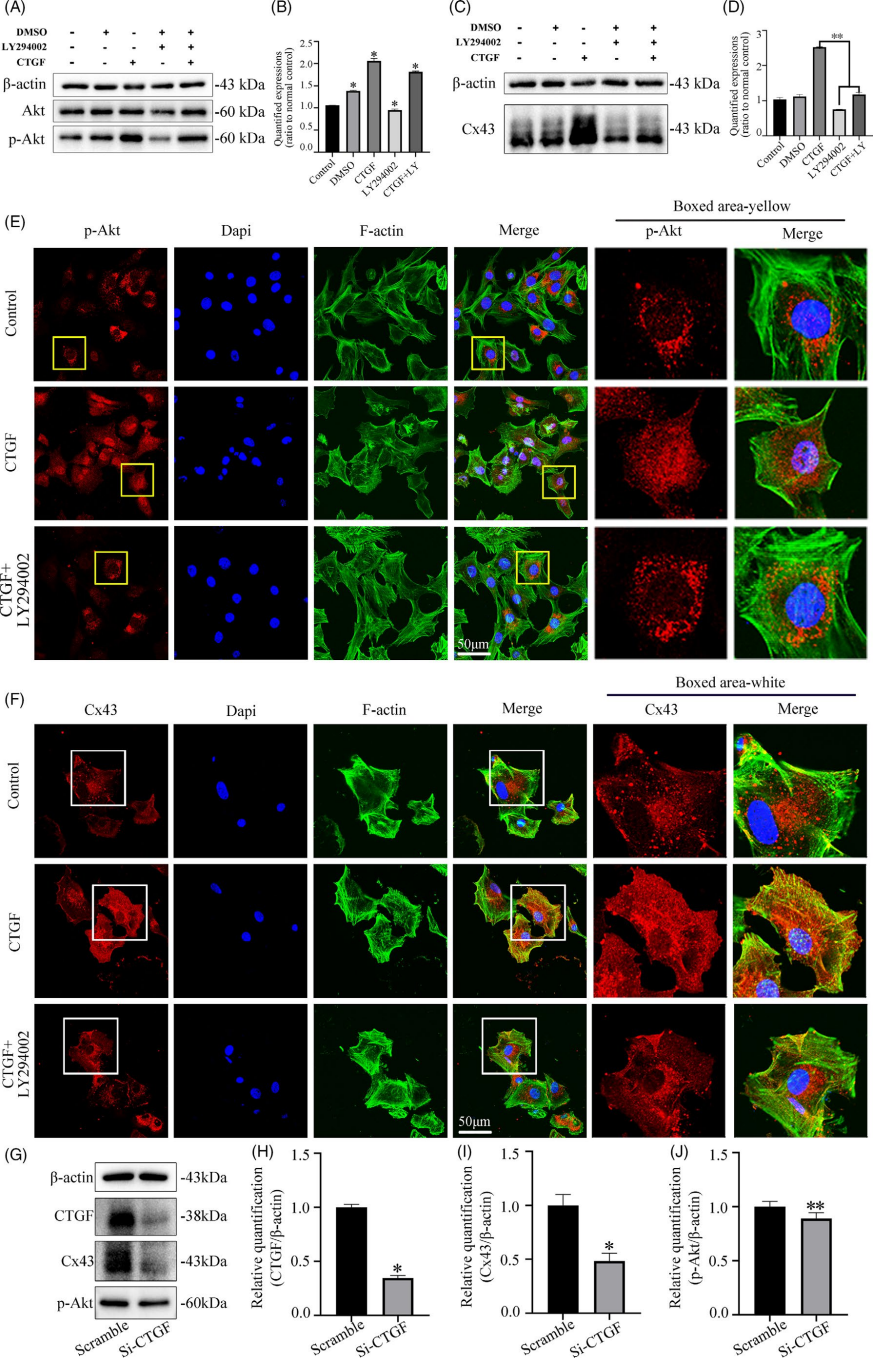
CTGF promotes Cx43 expression in chondrocytes via Pi3k/AKT signalling. A, Representative Western blot showing the expression changes of Akt signalling in chondrocytes induced by CTGF in the present of LY294002 (10 μmol/L). Samples were collected at 2 h after treatment. Data were derived from three independent experiments (n = 3). B, Quantification was done to show the protein changes of p‐Akt. The statistical analyses were based on three independent experiments (n = 3). *P < .05. C, Representative Western blot showing the expression changes of Cx43 in chondrocytes induced by CTGF (50 ng/mL) in the present of LY294002 (10 μΜ). Samples were collected at 12 h after treatment. Data were derived from three independent experiments (n = 3). D, Quantification was done to show the protein changes of Cx43. The statistical analyses were based on three independent experiments (n = 3). **P < .01. E, Representative IF staining by CLSM showing LY294002 attenuated CTGF‐induced p‐Akt activation after CTGF treatment (50 ng/mL). Cytoskeleton, green; Cx43, red; nuclei, blue. The results were derived from three independent experiments (n = 3). F, Representative IF staining by CLSM showing LY294002 attenuated CTGF‐induced Cx43 expression in the presence of CTGF (50 ng/mL). Cytoskeleton, green; Cx43, red; nuclei, blue. The results were derived from three independent experiments (n = 3). G, Representative Western blot showing the expression changes of CTGF, Cx43 and p‐Akt expression in chondrocytes after transfection with siRNA of CTGF at 48 h treatment. Data were based on three independent experiments (n = 3). H, Quantification was done to show the protein changes of CTGF in Figure G. The statistical analyses were based on three independent experiments (n = 3). *P < .05. I, Quantification was done to show the protein changes of Cx43 in Figure G. The statistical analyses were based on three independent experiments (n = 3). *P < .05. J, Quantification was done to show the protein changes of p‐Akt in Figure G. The statistical analyses were based on three independent experiments (n = 3). **P < .01

Further experiment by western blot confirmed that intense expression of p‐Akt protein induced by CTGF could be attenuated by the addition of LY294002 (Figure 4A&B). Accordingly, the increased Cx43 expression induced by CTGF can also be reduced by pretreatment with LY294002 at 2 hours (Figure 4C&D). Moreover, immunofluorescence was further performed to identify the expression of p‐Akt in chondrocytes induced by CTGF. The p‐Akt protein was located predominantly in the cytoplasm of no‐stimulated cells. Rather, p‐Akt accumulation was observed at the nuclear region at 2 hours with the stimulation with 50 ng/mL CTGF (Figure 4E). The enhanced p‐Akt expression induced by CTGF could be neutralized by addition of the PI3k inhibitor LY294002. (Figure 4E). Most importantly, immunofluorescence also showed CTGF alone increased the expression of Cx43, but this increase was significantly impaired at the presence of the inhibitor LY294002 (Figure 4F). Detailed research by western blot also revealed that inhibition of CTGF expression reduced both the Cx43 and p‐Akt expression at protein levels (Figure 4G‐J). Together, these results demonstrate the significant role of PI3K/AKT signalling in mediating CTGF‐induced Cx43 expression.

### 3.4 PI3K/Akt signalling shows its great importance in CTGF‐induced cell‐cell communication in chondrocytes

**FIGURE 5:**
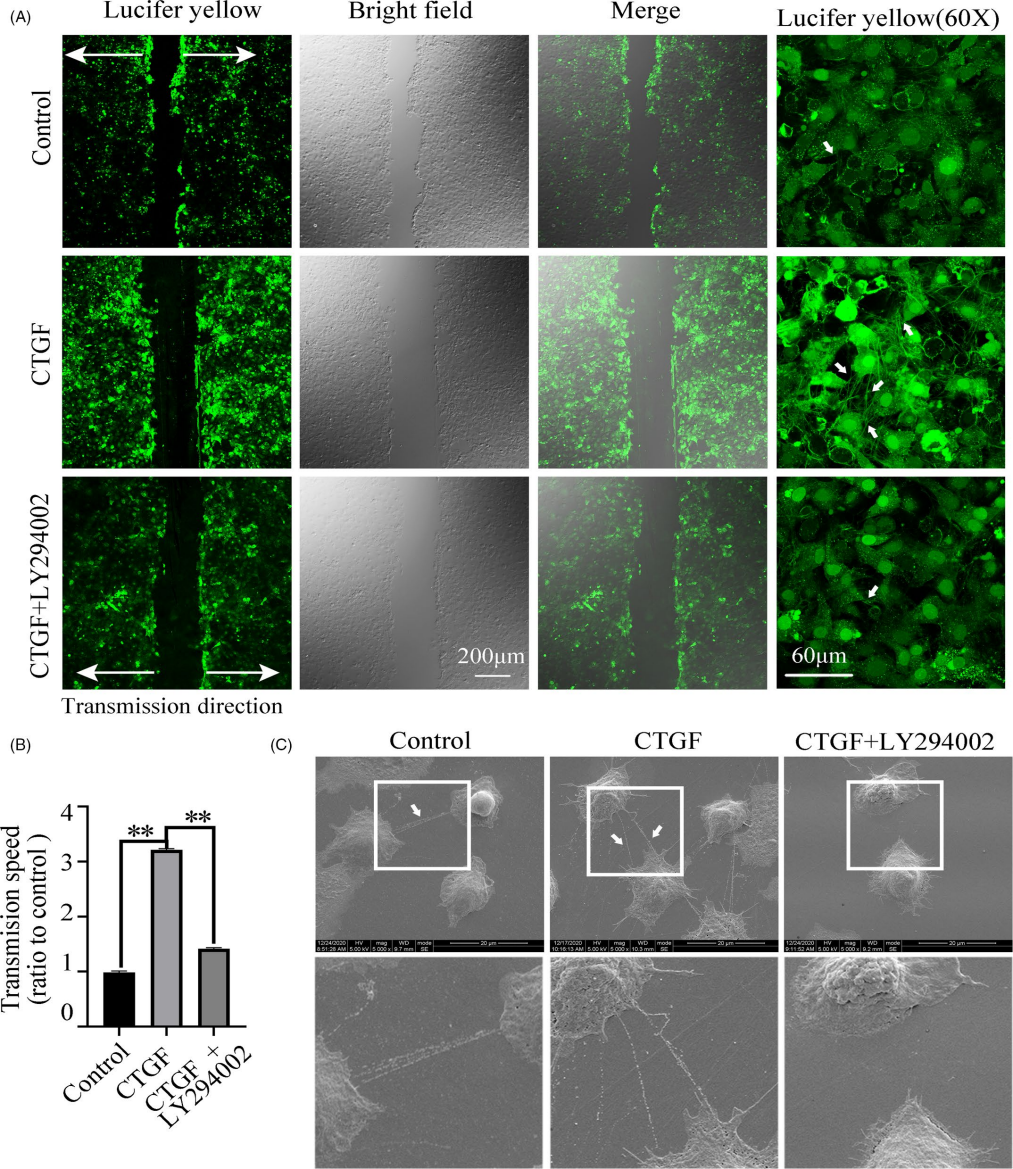
CTGF induces cell‐to‐cell communication in chondrocytes via Pi3k/AKT signalling. A, LY294002 attenuated CTGF‐induced transmission of Lucifer yellow in living chondrocytes. The concentration of LY294002 used was 10 μΜ. The concentration of CTGF was 50 ng/mL. The images were captured at 10 min after Lucifer yellow loading. White arrows represent the gap junctions formations. The results were derived from three independent experiments (n = 3). B, Quantification of the transmission speed of Lucifer yellow in living chondrocytes in Figure 5A. **P < .01. C, Representative SEM images showing LY294002 attenuated CTGF‐induced intercellular connection at 12 h treatment. The concentration of LY294002 used was 10 μΜ. The concentration of CTGF was 50 ng/mL. White boxed areas in the first line were zoomed in and displayed in the second one. The images were derived from three independent experiments (n = 3)

To further investigate whether the PI3K/Akt signalling could mediate the CTGF‐induced intercellular communication, we used scrape loading/dye transfer assay in living chondrocytes (Figure 5A). It was found that CTGF could increase the cell‐cell communication in the living chondrocytes by the promoting transmission speed of Lucifer yellow small molecule. But in the presence of inhibitor, LY294002, this increase induced by CTGF was greatly impaired. Quantification analysis further confirmed the result. (Figure 5B). Additionally, CLSM at 60x magnification showed that CTGF could promote more cell junctions formation and establish intricate communication network (white arrows in Figure 5A); this phenomenon also could be reverted by LY294002. SEM images also showed the cell junctions induced by CTGF could be reduced by LY294002 (Figure 5C).

## 4 DISCUSSION

**FIGURE 6:**
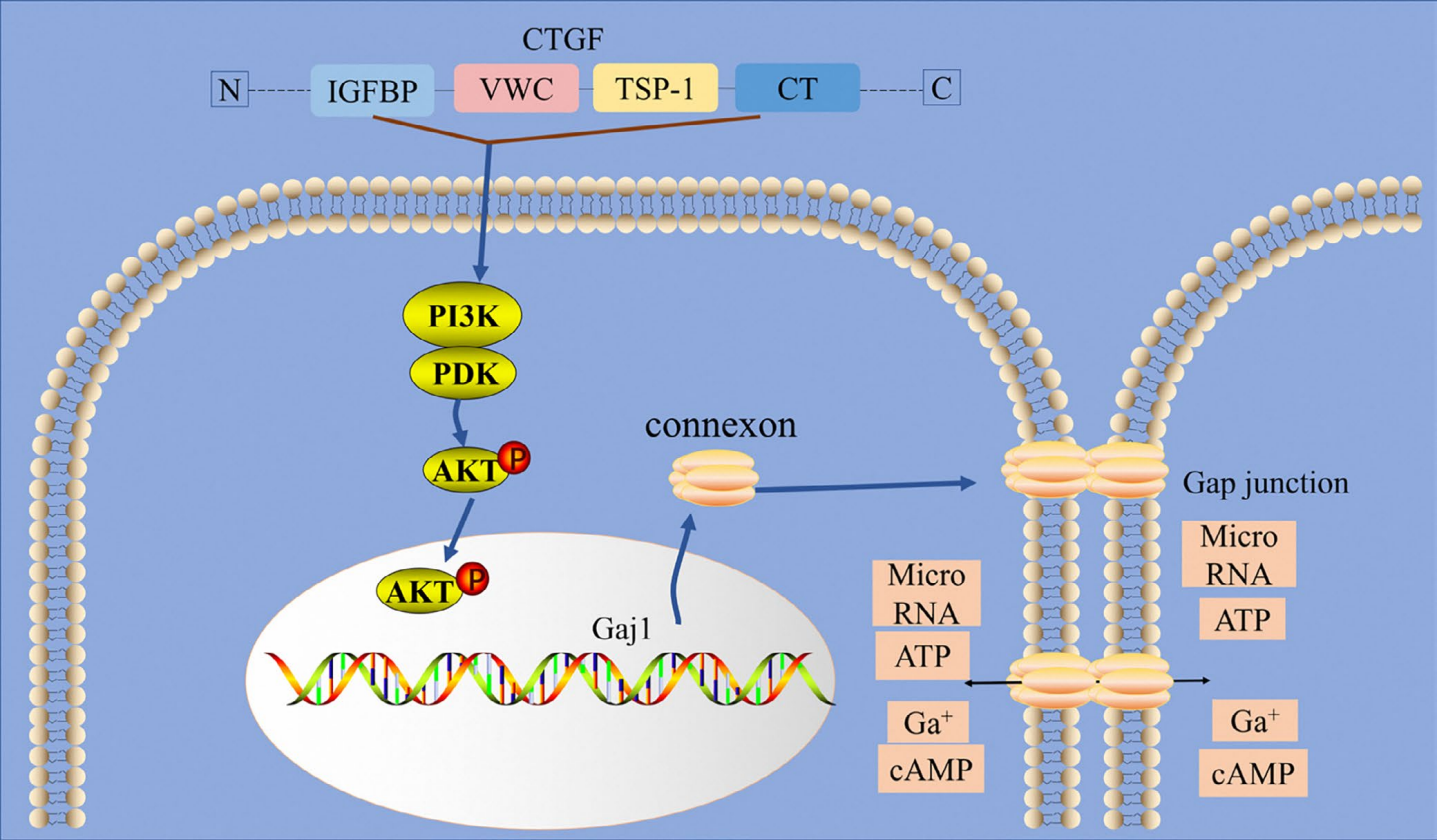
Schematic diagram illustrates the biomechanism of CTGF‐regulated Cx43 in chondrocytes. In brief, CTGF induced the PI3K/Akt signalling pathway to promote the expression of Cx43 and facilitate the gap junction formation

Articular chondrocytes exchange small molecules such as cyclic AMP (cAMP), ATP, microRNAs and calcium ions through forming functional gap junctions (GJs) and hemichannels such as Cx43, which plays vital roles in mechano‐transduction, differentiation, proliferation and metabolic homeostasis (Figure 6).^20^, ^23^, ^24^ Connexins in cartilage have been less well studied but Cx43 is the dominant connexins in chondrocytes.^18^, ^25^ Previous results of our study presented that the cell‐to‐cell communication could be regulated by the physical properties of the extracellular matrix^26^ and several growth factors.^19^, ^27^, ^28^ TGF‐β1 could up‐regulate cell‐cell communication both in chondrocytes and osteocytes.^19^, ^28^ CTGF acts as a downstream mediator of TGF‐β1 to induce mesenchymal cell condensation^29^ and ECM synthesis.^30^ CTGF is also a master regulator in the genesis of bone and cartilage.^31^ CTGF is also an important mediator of cardiac disease through up‐regulation of Cx43.^17^, ^32^ However, the relationship of CTGF with Cx43 expression and GJIC in chondrocytes remains unknown.

Our study provides evidence for the first time that CTGF stimulation increased intercellular communications between chondrocytes through increasing the formation of Cx43 gap junction channels and hemichannels. LY dye transfer experiments also confirmed CTGF‐treated group had strong ability to increase activity of GJIC. SEM showed CTGF could promote the formation of cell‐cell connection and Si‐CTGF reduced this phenomenon. More detailed results by confocal microscopy also showed that the distribution of Cx43 in chondrocytes also altered under the stimulation of CTGF, accompanied by the extensive expression of Cx43. Cx43 was accumulated in perinuclear region and then transported to the cell membrane to open hemichannels, which docked with nearby cells to form gap junctions. siRNA targeting CTGF in chondrocytes cloud suppress the intercellular communication and Cx43 expression. Other studies also confirmed that Cx43 was transferred from the endoplasmic reticulum to the Golgi, and then oligomerized in the trans‐Golgi network, and finally, transported to the plasma membrane along an intact cytoskeleton.^33^, ^34^ This phenomenon might indicate that Cx43 proteins were in a constant state of flux, providing a dynamic membrane domain in response to different stimuli, which is in consistent with previous study.^34^, ^35^ Align with previous study,^21^, ^36^ CTGF also effectively promoted chondrocytes proliferation and migration, which may owe to dual function of Cx43. That is, connexin proteins form gap junctions for the direct exchange of tissue homeostasis determinants between nearby cells and also act as an intercellular signalling reservoir by reversibly interacting with homeostasis determinants such as β‐catenin to modulate the production of cell growth and cell death.^37^ Consequently, we supposed that Cx43 not only form the gap junction but also directly interact with cell growth and cell death regulators under the stimulation of CTGF in chondrocytes. Further researches are required to verify this hypothesis.

The serine/threonine kinase protein kinase B (PKB)/Akt is a central player of various physiological functions such as metabolism, proliferation, survival, growth, angiogenesis, migration and invasion. Akt/PKB can be activated in a PI3K‐dependent manner with the stimulation of numerous growth factors, cytokines and hormones such as IGF‐1 and PTH.^38^, ^39^, ^40^ PI3K/AKT signalling is closely associated with Cx43. Inhibiting PI3K/ Akt signal transduction decreases Cx43 expression at mRNA and protein levels in MC3T3E1 cells.^39^ Previous studies have also revealed CTGF promoted cell proliferation, differentiation, extracellular matrix protein production, cell migration and actin cytoskeleton reorganization through PI3k/Akt pathway.^21^, ^41^, ^42^ To explore the potential mechanism of CTGF‐induced Cx43 expression, we examined whether PI3K/Akt signalling pathway was activated. In accordance with previous study,^41^, ^43^ Akt was phosphorylated and transported into the nucleus upon stimulation of CTGF in chondrocytes, where it regulated the activity of transcription, indicating the activation of the PI3K/Akt pathway (Figure 4E). Si‐CTGF in chondrocytes inhibited the expression of p‐Akt. Akt is initially synthesized on the endoplasmic reticulum, but translocated to the plasma membrane to be activated, and then repositions in various regions, including the cytosol and the cell nucleus,^44^ suggesting manifestation of differentially regulated phosphatase activity in these two compartments. From the results of confocal microscopy, p‐Akt was primarily concentrated in nucleus under the stimulation of CTGF.^45^ Accordingly, we speculate CTGF could play biological roles by inducing p‐Akt translocation in nucleus rather cytosol. Akt predominantly localizes in the cytoplasm, but it also translocates to the nucleus with treatment of growth factors.^46^ Nuclear PI3K/Akt signalling is also involved in cell survival, cell cycle progression control, cell differentiation, mRNA processing and exportation, DNA repair and tumorigenesis.^47^ But how Akt regulates cx43 expression remains unclear. There may be several potential mechanisms as below. Firstly, the transcription factor CREB (cAMP response element‐binding protein) is phosphorylated and activated by Akt on serine 133.^48^ Once phosphorylated on serine 133, CREB can induce transcription of connexin 43 gene.^48^ So we guess CTGF may induce Cx43 expression through PI3K/Akt signalling pathway to activate the CREB phosphorylation. Secondly, the half‐life of Cx43 mRNA was shortened in both LY294002‐treated cells and DN (dominant‐negative)‐Akt‐expressing‐MC3T3E1 cells, indicating Akt is associated the stability of mRNA.^39^ The AdenyMte/uridylate‐rich elements (ARE) are located in the 3' untranslated region (3'UTR), interacting with ARE‐binding proteins (ARE‐BPs), then regulating the mRNA stability of short‐lived mRNAs. Active Akt1 can directly phosphorylate ARE‐BPs, which could impair the decay of ARE containing transcripts.^49^, ^50^ Mouse and rat Cx43 3'UTRs contain four AREs and human Cx43 3'UTR contains five AREs, so Cx43 mRNAs may escape from the mRNA decay machinery by the phosphorylation of ARE‐BPs through PI3K/Akt activity. Consequently, CTGF may enhance PI3K/Akt activity to phosphorylate ARE‐BPs, then helping increase the stability of Cx43 mRNAs. At last, Akt could also phosphorylate Connexin43 protein on Ser373 to enlarge gap junctions by eliminating interaction between Cx43 and zona occludens‐1 (ZO‐1).^51^ More detailed researches need to be confirmed the above mechanism assumption. Additionally, our results showed that inhibition of PI3K/Akt pathway by LY294002 blocked the effects of CTGF on p‐Akt and Cx43‐gap junction activation, indicating the PI3K/Akt pathway mediated the stimulatory effect of CTGF on intercellular communication.

In conclusion, we demonstrated for the first time that CTGF promoted the Cx43 expression, gap junction formation as well as proliferation in chondrocytes with the involvement of PI3K/Akt signalling pathway (Figure 6). These findings suggested that CTGF actively participated in gap junction intercellular communication in chondrocytes.

## CONFLICT OF INTEREST

The authors report no conflicts of interest.

## AUTHOR CONTRIBUTIONS

Jing Xie and Shujuan Zou designed the experiments. Zuping Wu, Chenchen Zhou and Demao Zhang performed the experiments. Zuping Wu and Chenchen Zhou analysed and confirmed all data and prepared the manuscript. Quan Yuan evaluated all content including the manuscript draft and data reliability. All authors reviewed the manuscript, Jing Xie and Shujuan Zou made a final approval.

## Data Availability

No publicly available data or shared data are cited. All original data supporting the conclusion of the current study are available from the corresponding author on request.

## References

[1] Bhosale AM , Richardson JB . Articular cartilage: structure, injuries and review of management. Br Med Bull. 2008;87:77‐95. 18676397 10.1093/bmb/ldn025

[2] Hervé JC , Derangeon MI . Gap‐junction‐mediated cell‐to‐cell communication. Cell Tissue Res. 2013;352(1):21‐31. 22940728 10.1007/s00441-012-1485-6

[3] Goran S , Klaus W . Gap junctions and the connexin protein family. Cardiovasc Res. 2004;62(2):228‐232. 15094343 10.1016/j.cardiores.2003.11.013

[4] Baranova A , Ivanov D , Petrash N , et al. The mammalian pannexin family is homologous to the invertebrate innexin gap junction proteins. Genomics. 2004;83(4):706‐716. 15028292 10.1016/j.ygeno.2003.09.025

[5] Genetos DC , Kephart CJ , Zhang Y , Yellowley CE , Donahue HJ . Oscillating fluid flow activation of gap junction hemichannels induces ATP release from MLO‐Y4 osteocytes. J Cell Physiol. 2007;212(1):207‐214. 17301958 10.1002/jcp.21021 PMC2929812

[6] Jiang JX , Cherian PP . Hemichannels formed by connexin 43 play an important role in the release of prostaglandin E(2) by osteocytes in response to mechanical strain. Cell Commun Adhes. 2003;10(4–6):259‐264. 14681026 10.1080/cac.10.4-6.259.264

[7] Mayan MD , Carpintero‐Fernandez P , Gago‐Fuentes R , et al. Human articular chondrocytes express multiple gap junction proteins. Am J Pathol. 2013;182(4):1337‐1346. 23416160 10.1016/j.ajpath.2012.12.018 PMC3620397

[8] Schwab W , Hofer A , Kasper M . Immunohistochemical distribution of connexin 43 in the cartilage of rats and mice. Histochem J. 1998;30(6):413‐419. 10192540 10.1023/a:1003220225670

[9] Knight MM , Mcglashan SR , Garcia M , Jensen CG , Poole CA . Articular chondrocytes express connexin 43 hemichannels and P2 receptors ‐ a putative mechanoreceptor complex involving the primary cilium? J Anat. 2009;214(2):275‐283. 19207989 10.1111/j.1469-7580.2008.01021.x PMC2667885

[10] Graverand MPHL , Sciore P , Eggerer J , et al. Formation and phenotype of cell clusters in osteoarthritic meniscus. Arthritis Rheum. 2001;44:1808‐1818. 11508433 10.1002/1529-0131(200108)44:8<1808::AID-ART318>3.0.CO;2-B

[11] Arnott JA , Lambi AG , Mundy CM , Hendesi H , Popoff SN . The role of connective tissue growth factor (CTGF/CCN2) in skeletogenesis. Crit Rev Eukaryot Gene Expr. 2011;21(1):43‐69. 21967332 10.1615/critreveukargeneexpr.v21.i1.40 PMC3357314

[12] Brigstock D . The CCN family: a new stimulus package. J Endocrinol. 2003;178(2):169‐175. 12904165 10.1677/joe.0.1780169

[13] Perbal B . CCN proteins: multifunctional signalling regulators. Lancet. 2004;363(9402):62‐64. 14723997 10.1016/S0140-6736(03)15172-0

[14] De Winter P , Leoni P , De Winter P , et al. Connective tissue growth factor: Structure–function relationships of a mosaic, multifunctional protein. Growth Factors. 2008;26(2):80‐91. 18428027 10.1080/08977190802025602

[15] Cicha I , Goppelt‐Struebe M . Connective tissue growth factor: Context‐dependent functions and mechanisms of regulation. BioFactors. 2009;35(2):200‐208. 19449449 10.1002/biof.30

[16] Nishida T , Kubota S , Kojima S , et al. Regeneration of defects in articular cartilage in rat knee joints by CCN2 (Connective Tissue Growth Factor). J Bone Miner Res. 2004;19(8):1308‐1319. 15231019 10.1359/JBMR.040322

[17] Adam O , Lavall D , Theobald K , et al. Rac1‐induced connective tissue growth factor regulates connexin 43 and N‐cadherin expression in atrial fibrillation. J Am Coll Cardiol. 2010;55(5):469‐480. 20117462 10.1016/j.jacc.2009.08.064

[18] Gago‐Fuentes R , Carpintero‐Fernandez P , Goldring MB , Brink PR , Mayan MD , Blanco FJ . Biochemical evidence for gap junctions and Cx43 expression in immortalized human chondrocyte cell line: a potential model in the study of cell communication in human chondrocytes. Osteoarthritis Cartilage. 2014;22(4):586‐590. 24530659 10.1016/j.joca.2014.02.002

[19] Wang Q , Zhou C , Li X , et al . TGF‐β1 promotes gap junctions formation in chondrocytes via Smad3/Smad4 signalling. Cell Prolif. 2019;52(2):e12544. 30444057 10.1111/cpr.12544 PMC6495951

[20] Donahue HJ , Qu RW , Genetos DC . Joint diseases: from connexins to gap junctions. Nat Rev Rheumatol. 2017;14(1):42‐51. 29255213 10.1038/nrrheum.2017.204 PMC13464408

[21] Xing X , Li Z , Yu Z , Cheng G , Li D , Li Z . Effects of connective tissue growth factor (CTGF/CCN2) on condylar chondrocyte proliferation, migration, maturation, differentiation and signalling pathway. Biochem Biophys Res Commun. 2018;495(1):1447‐1453. 29198711 10.1016/j.bbrc.2017.11.190

[22] Deng Z , Liu Y , Wang C , Fan H , Ma J , Yu H . Involvement of PI3K/Akt pathway in rat condylar chondrocytes regulated by PTHrP treatment. Arch Oral Biol. 2014;59(10):1032‐1041. 24972187 10.1016/j.archoralbio.2014.04.012

[23] Zhang D , Li X , Pi C , et al. Osteoporosis‐decreased extracellular matrix stiffness impairs connexin 43‐mediated gap junction intercellular communication in osteocytes. Acta Biochim Biophys Sin (Shanghai). 2020;52(5):517‐526. 32286624 10.1093/abbs/gmaa025

[24] Liu W , Cui Y , Wei J , Sun J , Zheng L , Xie J . Gap junction‐mediated cell‐to‐cell communication in oral development and oral diseases: a concise review of research progress. Int J Oral Sci. 2020;12(1):17. 32532966 10.1038/s41368-020-0086-6 PMC7293327

[25] Mayan MD , Gago‐Fuentes R , Carpintero‐Fernandez P , et al. Articular chondrocyte network mediated by gap junctions: role in metabolic cartilage homeostasis. Ann Rheum Dis. 2015;74(1):275‐284. 24225059 10.1136/annrheumdis-2013-204244 PMC5500216

[26] Zhou C , Zhang D , Du W , Zou J , Li X , Xie J . Substrate mechanics dictate cell‐cell communication by gap junctions in stem cells from human apical papilla. Acta Biomater. 2020;107:178‐193. 32105834 10.1016/j.actbio.2020.02.032

[27] Liu X‐Y , Li X , Bai M‐R , et al. FGF‐7 dictates osteocyte cell processes through beta‐catenin transduction. Sci Rep. 2018;8(1):14792. 30287900 10.1038/s41598-018-33247-8 PMC6172271

[28] Liu W , Zhang D , Li X , et al. TGF‐β1 facilitates cell‐cell communication in osteocytes via connexin43‐ and pannexin1‐dependent gap junctions. Cell Death Discov. 2019;5:141. 31666990 10.1038/s41420-019-0221-3 PMC6814792

[29] Song JJ , Aswad R , Kanaan RA , et al. Connective tissue growth factor (CTGF) acts as a downstream mediator of TGF‐beta1 to induce mesenchymal cell condensation. J Cell Physiol. 2007;210(2):398‐410. 17111364 10.1002/jcp.20850

[30] Ihn H . Pathogenesis of fibrosis: role of TGF‐beta and CTGF. Curr Opin Rheumatol. 2002;14(6):681‐685. 12410091 10.1097/00002281-200211000-00009

[31] Takigawa M . CCN2: a master regulator of the genesis of bone and cartilage. J Cell Commun Signal. 2013;7(3):191‐201. 23794334 10.1007/s12079-013-0204-8 PMC3709051

[32] Tank J , Lindner D , Wang X , et al. Single‐target RNA interference for the blockade of multiple interacting proinflammatory and profibrotic pathways in cardiac fibroblasts. J Mol Cell Cardiol. 2014;66:141‐156. 24239602 10.1016/j.yjmcc.2013.11.004

[33] Nielsen MS , Axelsen LN , Sorgen PL , Verma V , Delmar M , Holstein‐Rathlou N‐H . Gap junctions. Compr Physiol. 2012;2(3):1981‐2035. 23723031 10.1002/cphy.c110051 PMC3821273

[34] Solan JL , Lampe PD . Spatio‐temporal regulation of connexin43 phosphorylation and gap junction dynamics. Biochim Biophys Acta Biomembr. 2018;1860(1):83‐90. 28414037 10.1016/j.bbamem.2017.04.008 PMC5640473

[35] Wang A , Xu C . The role of connexin43 in neuropathic pain induced by spinal cord injury. Acta Biochim Biophys Sin (Shanghai). 2019;51(6):555‐561. 31056639 10.1093/abbs/gmz038

[36] Nishida T , Kubota S , Nakanishi T , et al. CTGF/Hcs24, a hypertrophic chondrocyte‐specific gene product, stimulates proliferation and differentiation, but not hypertrophy of cultured articular chondrocytes. J Cell Physiol. 2002;192(1):55‐63. 12115736 10.1002/jcp.10113

[37] Vinken M , Decrock E , Leybaert L , et al. Non‐channel functions of connexins in cell growth and cell death. Biochim Biophys Acta. 2012;1818(8):2002‐2008. 21718687 10.1016/j.bbamem.2011.06.011

[38] Liu J , Liu M , Chen L . Novel pathogenesis: regulation of apoptosis by Apelin/APJ system. Acta Biochim Biophys Sin (Shanghai). 2017;49(6):471‐478. 28407045 10.1093/abbs/gmx035

[39] Bhattacharjee R , Kaneda M , Nakahama KI , Morita I . The steady‐state expression of connexin43 is maintained by the PI3K/Akt in osteoblasts. Biochem Biophys Res Commun. 2009;382(2):440‐444. 19285956 10.1016/j.bbrc.2009.03.044

[40] Yamamoto T , Kambe F , Cao X , Lu X , Ishiguro N , Seo H . Parathyroid hormone activates phosphoinositide 3‐kinase‐Akt‐Bad cascade in osteoblast‐like cells. Bone. 2007;40(2):354‐359. 17046344 10.1016/j.bone.2006.09.002

[41] Yosimichi G , Kubota S , Nishida T , et al. Roles of PKC, PI3K and JNK in multiple transduction of CCN2/CTGF signals in chondrocytes. Bone. 2006;38(6):853‐863. 16431170 10.1016/j.bone.2005.11.016

[42] Crean JKG , Finlay D , Murphy M , et al. The role of p42/44 MAPK and protein kinase B in connective tissue growth factor induced extracellular matrix protein production, cell migration, and actin cytoskeletal rearrangement in human mesangial cells. J Biol Chem. 2002;277(46):44187‐44194. 12218048 10.1074/jbc.M203715200

[43] Borgatti P , Martelli AM , Bellacosa A , et al. Translocation of Akt/PKB to the nucleus of osteoblast‐like MC3T3‐E1 cells exposed to proliferative growth factors. FEBS Lett. 2000;477(1–2):27‐32. 10899305 10.1016/s0014-5793(00)01758-0

[44] Toker A , Marmiroli S . Signalling specificity in the Akt pathway in biology and disease. Adv Biol Regul. 2014;55:28‐38. 24794538 10.1016/j.jbior.2014.04.001 PMC4062840

[45] Martelli AM , Tabellini G , Bressanin D , et al. The emerging multiple roles of nuclear Akt. Biochim Biophys Acta. 2012;1823(12):2168‐2178. 22960641 10.1016/j.bbamcr.2012.08.017

[46] Xuan Nguyen TL , Choi JW , Lee SB , et al. Akt phosphorylation is essential for nuclear translocation and retention in NGF‐stimulated PC12 cells. Biochem Biophys Res Commun. 2006;349(2):789‐798. 16956580 10.1016/j.bbrc.2006.08.120

[47] Davis WJ , Lehmann PZ , Li W . Nuclear PI3K signalling in cell growth and tumorigenesis. Front Cell Dev Biol. 2015;3:24. 25918701 10.3389/fcell.2015.00024 PMC4394695

[48] Salameh A , Krautblatter S , Karl S , et al. The signal transduction cascade regulating the expression of the gap junction protein connexin43 by beta‐adrenoceptors. Br J Pharmacol. 2009;158(1):198‐208. 19719782 10.1111/j.1476-5381.2009.00344.x PMC2795252

[49] Latifa AH , Chen CY , Giorgio C , et al. Identification of a set of KSRP target transcripts upregulated by PI3K‐AKT signalling. BMC Mol Biol. 2007;8:28. 17437629 10.1186/1471-2199-8-28 PMC1858702

[50] Schmidlin M , Lu M , Leuenberger SA , et al. The ARE‐dependent mRNA‐destabilizing activity of BRF1 is regulated by protein kinase B. The EMBO journal. 2004;23(24):4760‐4769. 15538381 10.1038/sj.emboj.7600477 PMC535089

[51] Park DJ , Wallick CJ , Martyn KD , Lau AF , Jin C , Warn‐Cramer BJ . Akt Phosphorylates Connexin43 on Ser373, a "Mode‐1" Binding Site for 14‐3‐3. Cell Commun Adhes. 2007;14(5):211‐226. 18163231 10.1080/15419060701755958 PMC2673107

